# Design, Preparation, and Evaluation of a Novel ^99m^TcN Complex of Ciprofloxacin Xanthate as a Potential Bacterial Infection Imaging Agent

**DOI:** 10.3390/molecules25245837

**Published:** 2020-12-10

**Authors:** Si’an Fang, Yuhao Jiang, Qianqian Gan, Qing Ruan, Di Xiao, Junbo Zhang

**Affiliations:** Key Laboratory of Radiopharmaceuticals, Ministry of Education, College of Chemistry, Beijing Normal University, Beijing 100875, China; 201531150045@mail.bnu.edu.cn (S.F.); 201921150103@mail.bnu.edu.cn (Y.J.); 201731150054@mail.bnu.edu.cn (Q.G.); 201931150055@mail.bnu.edu.cn (Q.R.); 202031150054@mail.bnu.edu.cn (D.X.)

**Keywords:** ciprofloxacin xanthate, quinolones, infection imaging, ^99m^Tc-radiolabeling

## Abstract

In order to seek novel technetium-99m bacterial infection imaging agents, a ciprofloxacin xanthate (CPF2XT) was synthesized and radiolabeled with [^99m^TcN]^2+^ core to obtain the ^99m^TcN-CPF2XT complex, which exhibited high radiochemical purity, hydrophilicity, and good stability in vitro. The bacteria binding assay indicated that ^99m^TcN-CPF2XT had specificity to bacteria. A study of biodistribution in mice showed that ^99m^TcN-CPF2XT had a higher uptake in bacterial infection tissues than in turpentine-induced abscesses, indicating that it could distinguish bacterial infection from sterile inflammation. Compared to ^99m^TcN-CPFXDTC, the abscess/blood and abscess/muscle ratios of ^99m^TcN-CPF2XT were higher and the uptakes of ^99m^TcN-CPF2XT in the liver and lung were obviously decreased. The results suggested that ^99m^TcN-CPF2XT would be a potential bacterial infection imaging agent.

## 1. Introduction

Public health advanced during the eighteenth and nineteenth centuries and with antibiotics discovered in the twentieth century, treatment of infection has been highly improved. However, at present, antibiotic abuse has led to the emergence of bacteria resistance, which limits the efficiency of antibiotics. Bacterial infection is one of the most common causes of morbidity and mortality in developing countries [1]. In the meantime, it is predicted that infections of antibiotic resistance would be the first cause of human death by 2050 [2]. Considering the patients carrying inflammation may have different disease processes occurring at the same time, the clinic assessments do not always find a clear pathogen, which leads to delay in treatment and antibiotic abuse. In that case, early detection of infections can allow for the timely and appropriate treatment of patients, avoiding the overuse of antibiotics [3].

Identifying and locating lesion sites of infection is a critical step in clinic treatment. Computed tomography (CT) and magnetic resonance imaging (MRI) are available for detecting infections. However, these imaging tools depend on anatomical changes that occur late in the disease process, so that in the early phase, they detect the infection foci inefficiently because obviously morphologic changes cannot be observed [4]. Compared to CT and MRI, nuclear medicine techniques such as Positron Emission Tomography (PET) and Single-Photon Emission Computed Tomography (SPECT) are based on physiochemical and biochemical changes in organs, which will be located in the early phase. Radiopharmaceuticals with high specificity can selectively concentrate at the site of infection, which leads to accurate detection of pathogens, and the rapid and suitable treatment of patients [1]. Currently, discriminating between infection and sterile inflammation has been of great significance. Various radiopharmaceuticals have been developed for detecting inflammation in humans, however, they have some limitations [5]. The development of new infection imaging radiopharmaceuticals is still needed in nuclear medicine.

Technetium-99m is extensively used in nuclear medicine, which is due to its in-house availability, low cost, decay characteristics, and the ability to conjugate with bioactive molecules through a bifunctional chelator [6]. Up to now, there have been several kinds of ^99m^Tc-labeled antimicrobial agents [7,8,9,10,11]. Among them, ^99m^Tc-ciprofloxacin has been widely evaluated by many groups in the world [12,13]. Compared to the radiolabeled leucocytes [14], ^99m^Tc-ciprofloxacin has more specificity for infection, lower cost of preparation, better imaging quality, and it can be prepared from a kit [15]. However, there are some limitations of ^99m^Tc-ciprofloxacin, such as low radiochemical yield and heating preparation [16]. In the structure of ciprofloxacin, the carbonyl and carboxyl groups are considered as necessary pharmacophores, which possibly coordinate with technetium-99m, therefore, labeling with ^99m^Tc would reduce the binding affinity to bacteria [17].

**Figure 1:**
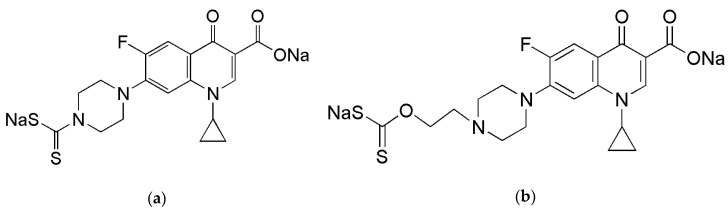
Chemical structures of CPFXDTC and CPF2XT: (**a**) ciprofloxacin dithiocarbamate, CPFXDTC; (**b**) ciprofloxacin xanthate.

[^99m^TcN]^2+^ core was found to conjugate well with ligands containing S atoms, such as dithiocarbamates [18]. Recently, our group has developed some ^99m^Tc-labeled antibiotic tracers. For example, we synthesized ciprofloxacin dithiocarbamate (CPFXDTC, as shown in Figure 1) and radiolabeled it with [^99m^TcN]^2+^ core. ^99m^TcN-CPFXDTC was easily prepared through a ligand-exchange reaction. In the biodistribution assay in bacteria-infected mice, the infection uptake was 3.21 ± 0.66% ID/g at 4 h post-injection. The abscess/muscle and abscess/blood ratios were 1.78 and 1.86. Compared to ^99m^Tc-ciprofloxacin, the abscess uptake and abscess/blood ratio were higher, but the abscess/muscle ratio was much lower. In addition, ^99m^TcN-CPFXDTC was lipophilic so the higher accumulation of radioactivity was found in the liver. The lung uptake was appreciable as well [18]. These limitations could affect the quality of infection imaging. Therefore, further research is needed to solve these problems.

As xanthate contains two sulfur atoms and can be easily labeled with technetium-99m, our group has recently reported some radiolabeled xanthates as potential imaging agents [19,20]. These backgrounds encourage us to synthesize a ciprofloxacin xanthate (CPF2XT, as shown in Figure 1) and evaluate the possibility of ^99m^TcN-CPF2XT as a potential bacterial infection imaging agent.

## 2. Results

### 2.1. Synthesis

**Scheme 1:**
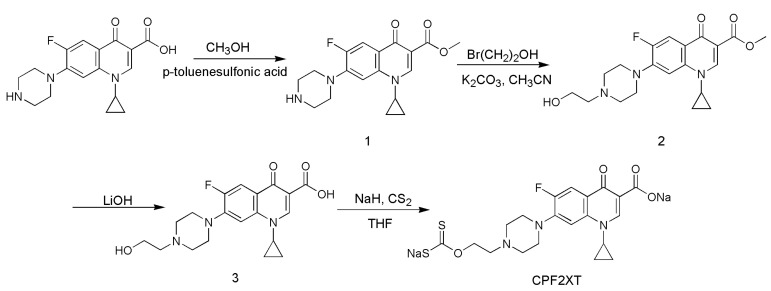
Synthesis of CPF2XT.

The reaction equation was shown in Scheme 1. CPF2XT was prepared by reacting the precursor *N*4′-2-hydroxyethylciprofloxacin (compound **3**) with carbon disulfide and NaH in THF. CPF2XT was characterized by ^1^H-NMR, ^13^C-NMR, and ESI-MS.

### 2.2. Radiolabeling

**Scheme 2:**
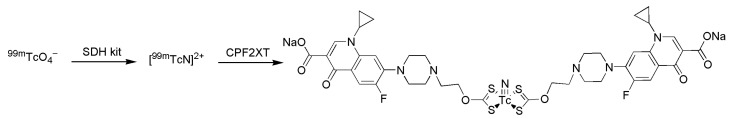
Preparation route and speculative structure of ^99m^TcN-CPF2XT.

^99m^TcN-CPF2XT was easily prepared in high yields through a ligand-exchange reaction, which was illustrated in Scheme 2. The radiochemical purity of the complex was determined by TLC. Results of TLC of ^99m^TcN-CPF2XT were as follows: in saline, ^99m^TcO_4_^−^ and ^99m^TcN-CPF2XT stayed at the origin, while [^99m^TcN]^2+^ was moved to the front. In acetonitrile, ^99m^TcO_4_^−^ was moved to 0.3–0.5 (R_f_ value), while [^99m^TcN]^2+^ and ^99m^TcN-CPF2XT remained at the origin.

In order to obtain the best labeling conditions, we optimized several parameters, such as pH of the solution, amount of ligand, reaction temperature, and incubation time. The labeling yield was over 90% when 5 mg of CPF2XT ligand was labeled with [^99m^TcN]^2+^ intermediate at pH 8–9 at room temperature for 30 min.

### 2.3. Physicochemical Properties Evaluation

#### 2.3.1. Stability Tests

In the reaction solution at room temperature after 6 h, the radiochemical purity of the complex was still more than 90%. In mouse serum at 37 °C, the radiochemical purity of the complex was over 90% as well. Nearly no decomposition of ^99m^TcN-CPF2XT was found, suggesting its great stability in vitro.

#### 2.3.2. Partition Coefficient

As compared to ^99m^TcN-CPFXDTC (log *P* = 1.02), the partition coefficients (log *P*) value of ^99m^TcN-CPF2XT was −0.80 ± 0.05. The result suggested ^99m^TcN-CPF2XT was hydrophilic while ^99m^TcN-CPFXDTC was lipophilic.

### 2.4. In Vitro Binding of ^99m^TcN-CPF2XT with Bacteria

**Figure 2:**
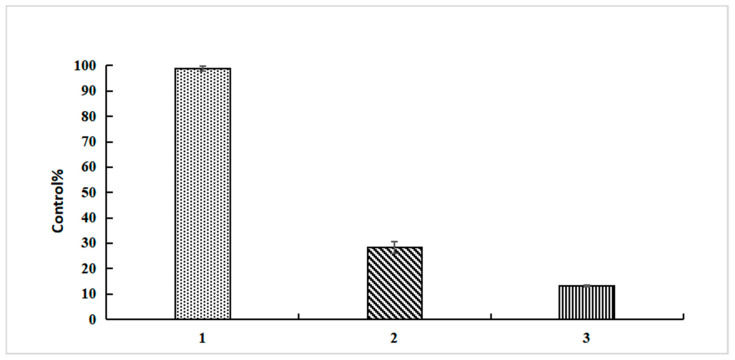
In vitro binding of ^99m^TcN-CPF2XT. Column 1: binding of ^99m^TcN-CPF2XT to bacteria, as the control group, the binding value was calculated as 100%; column 2: binding of ^99m^TcN-CPF2XT to bacteria competing with ciprofloxacin, and the data were shown as binding value/control group ratio; column 3: binding of ^99m^TcN-CPF2XT to bacteria competing with CPF2XT, and the data were shown as binding value/control group ratio.

The result of in vitro bacteria (*Staphylococcus. aureus*) binding study of ^99m^TcN-CPF2XT is shown in Figure 2. An excess of ciprofloxacin and CPF2XT were added respectively for competition. Columns 2 and 3 indicated that binding of the complex to the bacteria was significantly reduced. The values were separately decreased to 29.32% and 13.25% of the original value. It suggested that ^99m^TcN-CPF2XT was specifically binding to bacteria.

### 2.5. Biodistribution

Biological distribution results in mice bearing bacterial infection and turpentine-induced abscess were demonstrated in Table 1.

As seen in Table 1, in the bacteria-infected mice, the infection uptakes of ^99m^TcN-CPF2XT were 2.41 ± 0.37% ID/g at 2 h and 2.63 ± 0.49% ID/g at 4 h post-injection, suggesting it had a significant abscess uptake and good radioactivity retention in infection foci. The complex had low initial uptake in normal muscle, while relatively fast blood activity clearance was observed between 0.5 and 4 h post-injection, which led to the high abscess/muscle ratio and abscess/blood ratio.

In the meanwhile, the abscess uptake of ^99m^TcN-CPF2XT in mice with a turpentine-induced abscess was lower than that in bacteria-infected mice. The abscess uptake of ^99m^TcN-CPF2XT was 1.23 ± 0.13% ID/g at 4 h post-injection. The abscess/muscle and abscess/blood ratios were 2.51 and 1.70 at 4 h post-injection, which were also lower than those in bacteria-infected mice.

As for other organs, the high concentration in the kidney showed that the route of excretion was through the urinary system. The low value of radioactivity uptake in the stomach and thyroid indicated that the complex had good stability in vivo.

## 3. Discussion

Ciprofloxacin xanthate containing two sulfur atoms could conjugate with the [^99m^TcN]^2+^ core to obtain a stable complex in the form of ^99m^TcN(L)_2_ (L = bidentate ligand). [^99m^TcO_4_]^−^ and the SDH kit were mixed to form a [^99m^TcN]^2+^ intermediate. Then the ciprofloxacin xanthate was added to acquire ^99m^TcN-CPF2XT at room temperature with high labeling yield by a ligand-exchange reaction. In the meantime, ^99m^TcN-CPF2XT remains the pharmacophores of ciprofloxacin, which specifically bind to bacteria. Compared to ^99m^Tc-ciprofloxacin, the preparation of ^99m^TcN-CPF2XT does not require heating and purification, thus making it easier for clinical application.

In the in vitro bacteria binding assay, the binding efficiency of ^99m^TcN-CPF2XT to *S. aureus* with the corresponding ciprofloxacin xanthate for competition reduced by 86%, which showed its better specificity to bacteria. The biodistribution study indicated that ^99m^TcN-CPF2XT had good accumulation in infected foci (2.63% ID/g) at 4 h post-injection and the abscess/blood and abscess/muscle ratios were 4.78 and 4.04. However, the abscess uptake in mice with turpentine-induced abscess (1.23% ID/g) at 4 h post-injection was lower.

**Figure 3:**
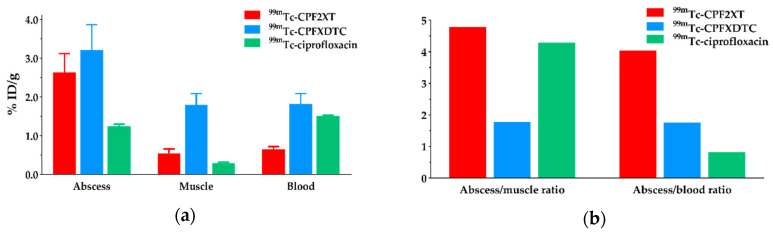
Comparison of biodistribution in mice of ^99m^TcN-CPF2XT, ^99m^TcN-CPFXDTC [18], and ^99m^Tc-ciprofloxacin [21]. (**a**) Infection uptake of the complexes at 4 h post-injection; (**b**) target/non-target ratio of the complexes at 4 h post-injection.

As shown in Figure 3, according to the results of biodistribution studies in bacteria-infected mice, the infection uptake of ^99m^TcN-CPF2XT (2.63% ID/g) was nearly two times as much as ^99m^Tc-ciprofloxacin (1.26% ID/g) at 4 h post-injection. Moreover, the abscess/blood ratio of ^99m^TcN-CPF2XT (4.04) was much higher than that of ^99m^Tc-ciprofloxacin (0.82). The abscess/muscle ratio of ^99m^TcN-CPF2XT was 4.78 at 4 h post-injection, while the abscess/muscle ratio of ^99m^Tc-ciprofloxacin was 4.28. Compared to ^99m^Tc-ciprofloxacin, ^99m^TcN-CPF2XT showed higher uptake in infected sections. For ^99m^Tc-ciprofloxacin, the carbonyl and carboxyl group could coordinate with technetium-99m, which led to the decreasing of binding affinity to bacteria. ^99m^TcN-CPF2XT had two sulfur atoms that could complex with the [^99m^TcN]^2+^ core to obtain a stable radiolabeled product. The pharmacophore of ciprofloxacin remained so that abscess uptake of ^99m^TcN-CPF2XT was higher than that of ^99m^Tc-ciprofloxacin. Compared to ^99m^TcN-CPFXDTC, the target/non-target ratio of ^99m^TcN-CPF2XT was much higher. At 4 h post-injection, the abscess/blood ratio and abscess/muscle ratio were 4.78 and 4.04, while the corresponding data of ^99m^TcN-CPFXDTC were 1.78 and 1.76. As for other non-target organs, the radioactive accumulations of the lung (21.11 ± 6.80% ID/g) and the liver (34.65 ± 5.93% ID/g) of ^99m^TcN-CPFXDTC were appreciable, while the lung (3.09 ± 0.24% ID/g) and liver (7.02 ± 0.63% ID/g) uptakes of ^99m^TcN-CPF2XT were much lower. According to the partition coefficient data of these two, ^99m^TcN-CPFXDTC was lipophilic, while ^99m^TcN-CPF2XT was hydrophilic. The hydrophilicity of ^99m^TcN-CPF2XT possibly caused the lower uptake of the non-targets. In that case, ^99m^TcN-CPF2XT could obtain a higher quality of abdominal images than ^99m^TcN-CPFXDTC. Comparing the results of biodistribution of infected and inflammation mice, we found a significant difference between the uptake in bacteria-infected and inflammation foci. The results suggested that ^99m^TcN-CPF2XT had the potential to distinguish infection from sterile inflammation. It should be noted that the bacterial infection uptake of ^99m^TcN-CPF2XT was not high enough, thus possibly making it insufficient to obtain satisfactory imaging results. Further studies should be conducted to verify this speculation.

In the discovery of ^99m^Tc imaging agents, most ^99m^Tc (V) complexes are related to [^99m^TcO]^3+^ core and [^99m^TcN]^2+^ core. The [^99m^TcO]^3+^ core is isoelectronic with [^99m^TcN]^2+^, which has the ability to complex with the ciprofloxacin xanthate to form a stable labeled complex. By comparison, we prepared ^99m^TcO-CPF2XT through a ligand-exchange reaction by mixing CPF2XT and [^99m^TcO_4_]^−^ with the GH kit [22]. The log *P* value of ^99m^TcO-CPF2XT is −0.12 ± 0.05 and the bacteria binding value reduced by 58% after the excess of CPF2XT was added as an inhibitor. The uptake of ^99m^TcO-CPF2XT in infected mice was 1.63 ± 0.23% ID/g at 4 h post-injection. In the inflammation mice, the abscess uptake was 1.44 ± 0.13% ID/g at 4 h post-injection. There was no significant difference between the infected and inflammation mice, suggesting ^99m^TcO-CPF2XT was non-specific to bacteria. The finding suggested different ^99m^Tc cores may have a great impact on the properties of ^99m^Tc-labeled radiopharmaceuticals. The incorporation of the ^99m^TcN core into the ciprofloxacin xanthate ligand can improve the biological features for infection imaging.

## 4. Materials and Methods

Ciprofloxacin was purchased from J&K chemical. Carbon disulfide was purified by distillation before use. Succinic dihydrazide (SDH) kit was acquired from Beijing Shihong Pharmaceutical Center, Beijing Normal University, China. All other agents were of reagent grade and were used with no further purification. ^99^Mo/^99m^Tc generator was purchased from Atomic High Tech Co. Ltd., Beijing, China. The NMR spectrum was recorded on a 600 MHz JNM-ECS spectrophotometer (JEOL, Tokyo, Japan). The ESI-MS spectrum was recorded on an LC–MS Shimadzu 2010 series.

### 4.1. Synthesis of CPF2XT

The precursor *N*4′-2-hydroxyethylciprofloxacin (compound **3**) was prepared according to a previously reported method [23]. A total of 0.300 g of compound **3** and 0.043 g of NaH were mixed in 20 mL of THF. Next, 0.5 mL of carbon disulfide was added to the solution. The mixture was stirred for 2 h in an ice water environment and then continued overnight at room temperature. Deionized water was added to remove the excess NaH. The solvent was removed under reduced pressure and the residue was recrystallized from methanol/diethyl ether to give CPF2XT (yellow solid, 0.258 g, 65.15%). ^1^H-NMR (D_2_O, δ): 8.33 (s, 1H), 7.66 (d, *J* = 13.4 Hz, 1H), 7.36 (d, *J* = 7.2 Hz, 1H), 3.70–3.60 (m, 2H), 3.45 (s, 1H), 3.13 (s, 4H), 2.61 (s, 4H), 2.57–2.46 (m, 2H), 1.25–1.13 (m, 2H), 0.95 (s, 2H). ^13^C-NMR (D_2_O, δ): 181.56, 175.43, 172.64, 171.14, 154.13, 152.49, 146.83, 144.03, 138.61, 122.27, 116.99, 111.53, 106.68, 58.96, 58.24, 52.28, 49.48, 34.82, 23.37, 7.48. ESI-HRMS: calculated for m/z C_20_H_20_FN_3_NaO_4_S_2_ [M − Na]^−^ 472.0782, found 472.0770.

### 4.2. Radiolabeling of ^99m^TcN-CPF2XT and Quality Control Assay

^99m^TcN-CPF2XT was prepared by a ligand-exchange reaction: 1 mL of saline with ^99m^TcO_4_^−^ was added to an SDH kit. The solution was prepared at room temperature for 20 min to obtain the [^99m^TcN]^2+^. Then, 5 mg of CPF2XT was dissolved in 1 mL of saline was added to the mixture. The reaction solution was kept at room temperature for 30 min.

The radiochemical purity of ^99m^TcN-CPF2XT was evaluated by thin-layer chromatography (TLC). TLC was performed by using a polyamide strip as a stationary phase, and saline and acetonitrile as mobile phases, respectively.

### 4.3. Stability Study

The stability of ^99m^TcN-CPF2XT was evaluated by measuring the radiochemical purity (RCP) of the complex. RCP was checked by TLC in the mixture at room temperature for 6 h and in mouse serum at 37 °C for 6 h.

### 4.4. Partition Coefficient Measurement

The partition coefficient was measured as follows: the radiolabeled complex was mixed with an equal volume of phosphate buffer (0.025 mol/L, pH 7.4) and 1-octanol. The mixture was vortexed at room temperature for 1 min and centrifuged at 5000 r/min for 5 min afterward. Counts in 0.1 mL of the organic and aqueous layers were calculated with a well γ-counter. The partition coefficient value was calculated by the equation: *P* = (cpm in 1-octanol)/(cpm in buffer). The final partition coefficient was expressed as log *P* [24].

### 4.5. In Vitro Bacteria Binding Study

In vitro bacteria binding of ^99m^TcN-CPF2XT was evaluated by using a previously reported method [25]. A total of 0.1 mL of 3.7 MBq ^99m^TcN-CPF2XT solution and 0.1 mL PBS (pH 7.4) containing about 1 × 10^8^ *S. aureus* were added into a test tube with 0.8 mL of saline. The mixture was then incubated for 1 h at 37 °C. Afterward, they were centrifuged at 2000 r/min for 5 min. The pellets were resuspended in 1 mL PBS (pH 7.4) and recentrifuged. The removed supernatant and the bacteria pellets were collected and the radioactivities of those were determined with a well γ-counter, respectively. The bacteria binding value was calculated by the equation as follows: Bacteria binding% = (cpm in precipitate − cpm in background)/(cpm in precipitate − cpm in background + cpm in supernatant) × 100%. The background tube was the incubations without bacteria added.

For the sake of determining the specificity of ^99m^TcN-CPF2XT binding to bacteria, ciprofloxacin and CPF2XT were used as inhibitors. The bacteria were preincubated with 10 mg/mL ciprofloxacin or CPF2XT for 1 h at 37 °C. Then the ^99m^TcN-CPF2XT complex was added and incubated for 1 h at 37 °C. The bacteria binding value was calculated as above. The results were expressed as the mean ± SD.

### 4.6. Biodistribution Study in Bacterial Infected Mice

Animal studies were performed in accordance with the Regulations on Laboratory Animals of Beijing Municipality and the guidelines of the Ethics Committee of Beijing Normal University. A 0.1 mL suspension (1 × 10^8^/mL *S. aureus*) was injected into the left thigh muscle of the Kunming female mice weighing 18–22 g. About three to five days later, 0.1 mL of the complex (7.4 × 10^5^ Bq) was injected intravenously into each mouse via the tail vein. The mice were sacrificed at 0.5, 2, and 4 h post-injection. The infected section, normal muscle in the right thigh, blood, and other organs of interest were kept, weighed, and determined for radioactivity. The results were expressed as a percentage of injected dose per gram of tissue (% ID/g).

### 4.7. Biodistribution Study in Turpentine-Induced Abscess Mice

A total of 0.1 mL of turpentine was injected into the left thigh muscle of the Kunming female mice weighing 18–22 g. About seven days later, 0.1 mL of the complex (7.4 × 10^5^ Bq) was injected intravenously into each mouse via the tail vein. The mice were sacrificed at 4 h post-injection. The abscess, normal muscle in the right thigh, blood, and other organs of interest were kept, weighed, and determined for radioactivity. The results were expressed as a percentage of injected dose per gram of tissue (% ID/g).

## 5. Conclusions

In this work, ciprofloxacin xanthate (CPF2XT) was successfully prepared, and it was labeled with the ^99m^TcN precursor to obtain ^99m^TcN-CPF2XT with high yields. ^99m^TcN-CPF2XT showed hydrophilicity, good stability, and specificity to bacteria. The biodistribution results in mice suggested ^99m^TcN-CPF2XT exhibited high infection uptake and target-to-non target ratio. Compared to the results in turpentine-induced abscess mice, the complex was able to distinguish infection from inflammation. By comparison, the target/non-target ratio of ^99m^TcN-CPF2XT was higher than that of ^99m^TcN-CPFXDTC and the liver and lung uptakes of the former were much lower than those of the latter. In the present study, ^99m^TcN-CPF2XT showed its potential as a good bacterial infection imaging tracer, justifying further investigations.

## Figures and Tables

**Table 1:** Biodistribution of ^99m^TcN-CPF2XT in mice (% ID/g ± SD).

	Mice with Bacterial Infection (n = 5)			Mice with Turpentine-Induced Abscess (n = 5)
Tissue	0.5 h	2 h	4 h	4 h
Abscess	2.12 ± 0.27	2.41 ± 0.37	2.63 ± 0.49	1.23 ± 0.13
Muscle	0.82 ± 0.38	0.57 ± 0.14	0.55 ± 0.11	0.49 ± 0.16
Blood	1.78 ± 0.39	0.69 ± 0.09	0.65 ± 0.07	0.72 ± 0.07
Liver	13.12 ± 2.41	6.83 ± 0.88	7.02 ± 0.63	9.58 ± 0.82
Lung	4.42 ± 1.15	3.21 ± 1.08	3.09 ± 0.24	3.52 ± 0.16
Kidney	18.23 ± 2.42	16.28 ± 0.88	13.56 ± 0.61	14.27 ± 1.08
Stomach	3.12 ± 1.28	2.24 ± 1.00	2.55 ± 0.67	2.95 ± 0.53
Thyroid (%ID)	0.03 ± 0.01	0.02 ± 0.01	0.03 ± 0.01	0.04 ± 0.02
T/N ^1^	2.58	4.22	4.78	2.51
T/B ^2^	1.19	3.49	4.04	1.70

^1^ T/N = abscess/muscle ratio, ^2^ T/B = abscess/blood ratio.
